# Visual acuity assessment of central retinal artery occlusion patients with or without paracentral acute middle maculopathy via OCT-A

**DOI:** 10.1186/s12886-023-03151-5

**Published:** 2023-10-13

**Authors:** Hongxia Gong, Bin Wu, Shiyong Xie

**Affiliations:** 1 Department of Integrated Traditional Chinese and Western Medicine Ophthalmology, Tianjin Key Laboratory of Ophthalmology and Visual Science, Tianjin Eye Hospital, 300010 Tianjin, People’s Republic of China; 2 Department of Visual Function Division, Tianjin Key Laboratory of Ophthalmology and Visual Science, Tianjin Eye Hospital, 300010 Tianjin, People’s Republic of China

**Keywords:** Central retinal artery occlusion, Fundus fluorescein angiography, Optical coherence tomography angiography, Paracentral acute middle maculopathy, Visual acuity

## Abstract

**Purpose:**

The association between paracentral acute middle maculopathy (PAMM) and visual acuity in patients with central retinal artery occlusion (CRAO) is still unclear. The present study investigated the visual acuity of CRAO patients with and without PAMM.

**Methods:**

CRAO patients with PAMM or without PAMM were included. Optical coherence tomography angiography (OCT-A) was used to record the macular retinal thickness and density of shallow and deep vessels. The Best-corrected visual acuity (BCVA) was converted to a logarithm of the minimum angle of resolution (LogMAR) for statistical analysis.

**Results:**

There were 34 CRAO patients with PAMM (43.13%), other 30 CRAO patients without PAMM (46.87%). Compared with the no-PAMM group, PAMM group had better LogMAR BCVA (1.48 (0.49, 1.85) Vs. 1.85 (1.70, 1.96), *P* < 0.01). There was also a significant difference in retinal thickness of the central macular sulcus (328.00 (304.50–332.25) Vs. 352.50 (311.75–420.50), *P* = 0.01). A significant correlation between LogMAR BCVA and macular retinal thickness was found (r = 0.42; *P* < 0.01).

**Conclusion:**

CRAO patients with PAMM had significantly better visual acuity and less macular edema. OCT-A can be used to distinguish different levels of damage due to CRAO.

## Introduction

Central retinal artery occlusion (CRAO) is a common ophthalmic emergency that often leads to severe vision loss and permanent visual impairment. More apparent retinal damage occurs with longer durations of ischemia [1]. It was reported the annual incidence of CRAO was about 1.9 in 100,000 people [2]. CRAO is usually embolic in origin [1]. The Factors associated with CRAO include cigarette smoking, body mass index, hypertension, diabetes, high serum lipid levels, coagulopathy, and cardiac disease, including atrial fibrillation [3]. Typically, CRAO manifests as a sudden, painless, monocular loss of visual acuity and peripheral vision [4]. The presenting visual acuity could vary widely, and the patient may or may not have readily visible fundus abnormalities [3]. Before progression to a more complete CRAO, paracentral acute middle maculopathy (PAMM) may be the first sign to be noted. With the advent of optical coherence tomography angiography (OCT-A), it is possible to perform depth-resolved imaging and detailed visualization of the retinal capillary system in vivo. OCT-A is helpful in detecting PAMM. PAMM is not an isolated phenomenon but a common sign of several ocular diseases or even systemic conditions [5–7]. PAMM may be caused by ischemia of the intermediate and deep capillary systems, which are responsible for blood supply to the middle retina [8, 9]. It was reported that approximately 22.38% (32/143) of the Chinese patients with CRAO showed PAMM on spectral domain optical coherence tomography (SD-OCT) [10]. The association between PAMM and visual acuity in patients with CRAO is still unclear. In this study, we used OCT-A to analyze CRAO in patients with and without PAMM.

## Methods

### Patients

Thirty-four CRAO patients with PAMM and 30 CRAO patients without PAMM were included in this retrospective study from June 2016 to June 2020. The period from symptom onset to treatment was less than seven days for all the patients. All patients underwent ocular examination, including the best-corrected visual acuity (BCVA) test, slit-lamp microscopy, indirect ophthalmoscopy, intraocular pressure examination, fundus photography, and OCT-A. The macular retinal thickness on OCT-A was recorded, and the fundus fluorescein angiography (FFA) examination was performed within three days of the visit. The clinical diagnosis was based on the reported criteria [1, 11]. The exclusion criteria were as follows: (1) patients who had ciliary retinal arteries; (2) FFA showing a standard circulation time; (3) history of ocular trauma, surgery, or other ocular diseases (e.g., diabetic retinopathy, posterior uveitis, ocular hypertension, and glaucoma, etc.) or systemic diseases or systemic diseases that may interfere with our observations; (4) refusal to undergo FFA or other tests for this study; (5) equivalent spherical lens prescription >3.00 D; and (6) refractive interstitial opacities affecting this observation. The study was approved by the Ethics Committee of Tianjin Eye Hospital (No. 202,079). All patients provided informed consents.

### Ophthalmic examination

Visucam PRO NM fundus photography system (Carl Zeiss Meditec, Inc., Germany) was used to perform the fundus color photography. After mydriasis by Tropicamide, FFA was performed with Heidelberg retinal tomography (Heidelberg Engineering, Heidelberg, Germany), observing for 20 minutes. OCT-A examination of the macular area was conducted by Vue software from RTVue-XR Avanti (Optovue, Inc., Fremont, CA, USA). The 6 mm × 6 mm scan mode was selected, with transverse and longitudinal scanning for 3 seconds. Chosen clear images to store on the computer. All procedures were performed by a skilled physician. Systematic default stratification was used [12], i.e., a superficial capillary layer (internal limiting membrane-inner plexiform layer; ILM-IPL), deep retinal capillary layer (inner plexiform layer-outer plexiform layer; IPL-OPL), outer retinal layer (outer plexiform layer-Bruch’s membrane; OPL-BRM), and choroidal capillary layer (BRM-BRM + 30 µm). The blood vessels of the superficial retinal, deep retinal capillary layers, and the corresponding en face and B-scan images were observed. The system was equipped with software for the retinal thickness of the central macular sulcus and vascular density. All instruments used were calibrated by experienced technicians. The BCVA was converted to a logarithm of the minimum angle of resolution (LogMAR) for statistical analysis (LogMAR = Log (1/fractional visual acuity); for ‘index’ and ‘manual’ visual acuity conversions, please refer to the literature for details [13].

### Statistical analysis

Statistical analysis was performed using SPSS version 26.0 (SPSS, Chicago, IL, USA). Continuous data with a normal distribution were presented as means ± standard deviation and analyzed using the independent sample *t*-test; otherwise, they were presented as median (IQR) and analyzed using the Mann–Whitney *U* test. Categorical data were presented as n (%) and analyzed using the chi-square test. Spearman correlation was employed to analyze the correlation between indicators. *P* value < 0.05 was considered statistically significant.

## Results

### Fundus examination results

**Fig. 1:**
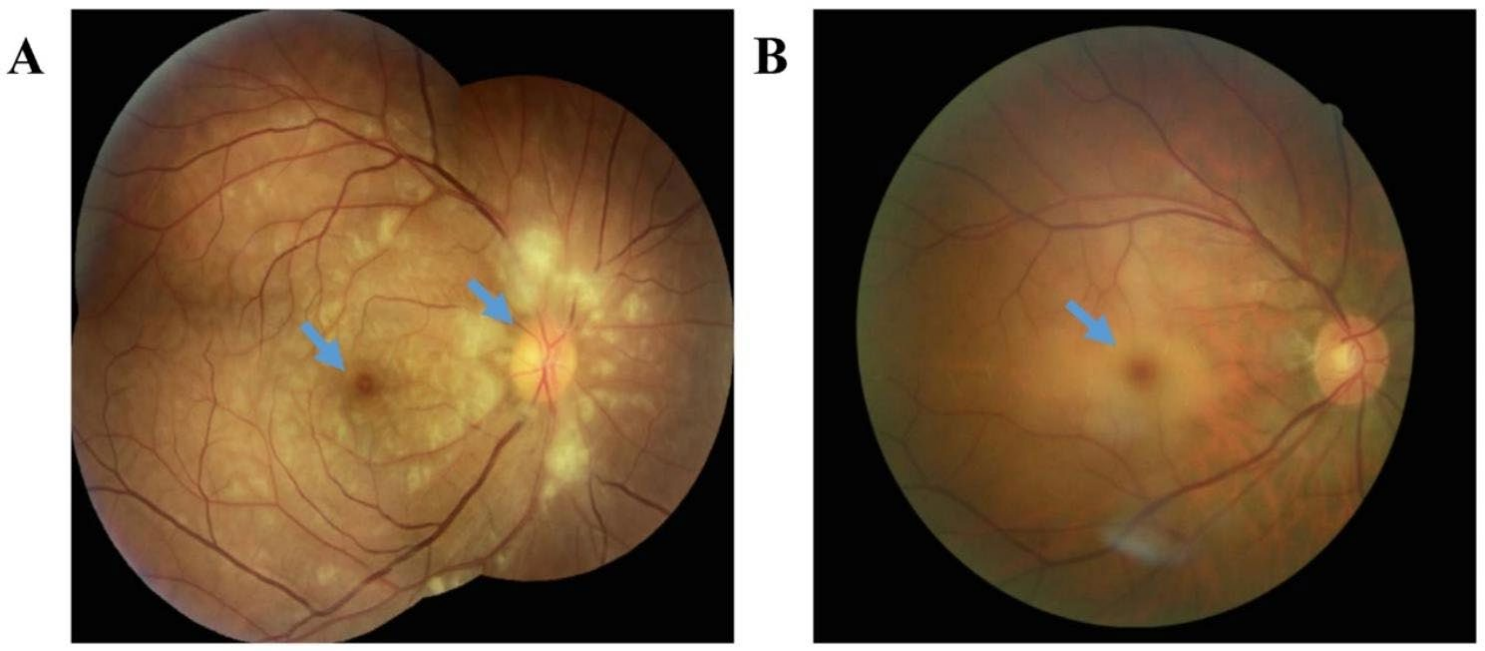
The fundus images. **A**. As shown by the arrow in the fundus image of PAMM patient, there were several cotton spots around the optic disc and retina and PAMM-like changes in macular area. **B**. As shown by the arrow in the fundus image of non-PAMM patient, the retina was pale and edematous based on fundus photography, and the macula was cherry red. PAMM: paracentral acute middle maculopathy

For the fundus examination, the boundary of the optic disc of CRAO patients with PAMM may or may not be sufficiently clear. The color was slightly lighter, and either diffuse posterior pole retinal edema or several retinal cotton spots were observed, accompanied by a small amount of bleeding in some cases (Fig. 1A). Differently, the optic disc boundary in the non-PAMM group was clear, and the color was light only in some cases. Posterior pole retinal pale edema was prominent, accompanied by retinal cotton spots, bleeding, or cherry-red macular spots in some cases (Fig. 1B).

### FFA examination results

**Fig. 2:**
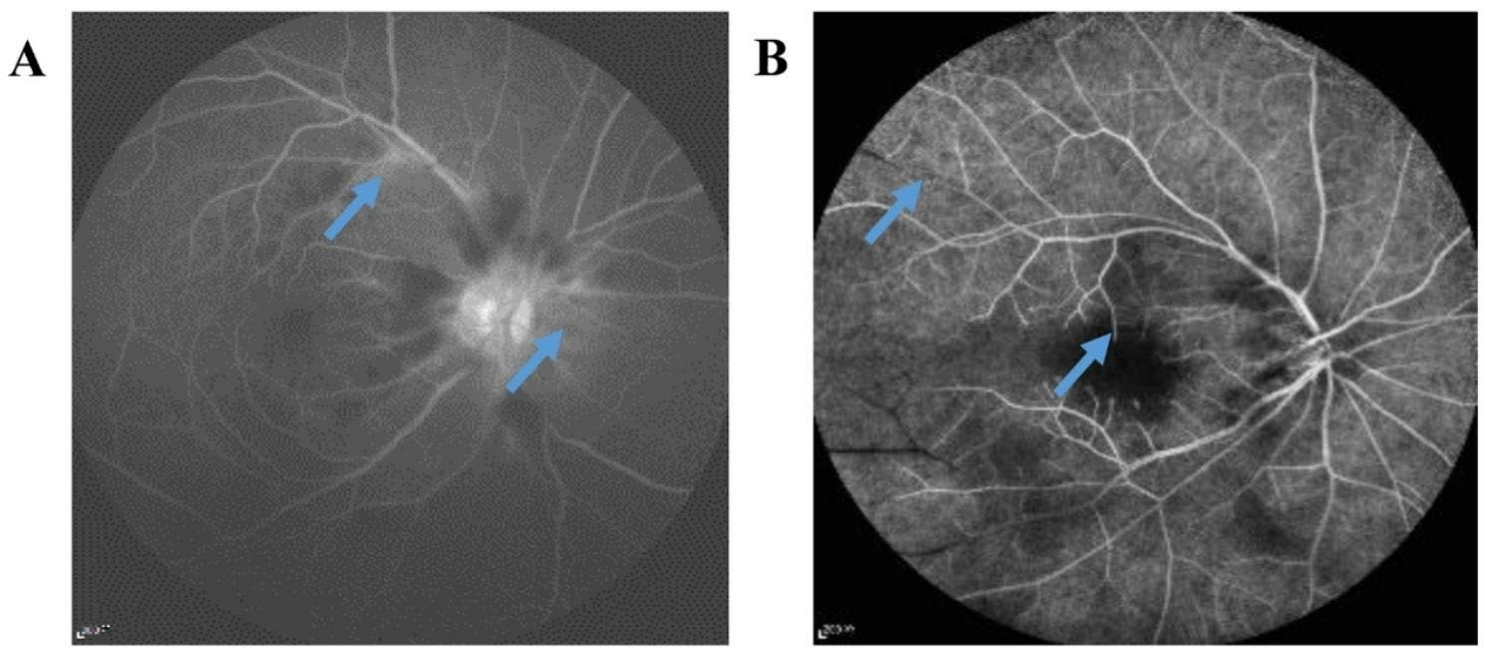
The FFA images. **A**. In the FFA image of PAMM patient, the arrows indicated there was a small amount of leakage in the optic disc and some retinal vessels in the late stage. **B**. In the FFA image of non-PAMM patient, the arrows indicated there were no peripheral retina filling and no perfusion in the macular region. FFA: fundus fluorescein angiography; PAMM: paracentral acute middle maculopathy

With a delayed retinal arteriovenous filling, staining or leakage of the optic disc and vessel wall was observed in the late stage in both the PAMM and non-PAMM groups (Fig. 2). Most non-PAMM patients (22/30, 73.33%) showed high fluorescence of the optic disc and no perfusion of the peripheral retina or macula. In six non-PAMM patients (6/30, 20.00%), only a small amount of filling of the optic disc and peripapillary retinal vessels was observed in the late stage, and most of the retinal vessels were not filled. A tiny fraction of patients (2/30, 6.67%) showed delayed arteriovenous filling, and no obvious hypoperfusion of retinal vessels in the later stage was found.

### OCT-A examination results

**Fig. 3:**
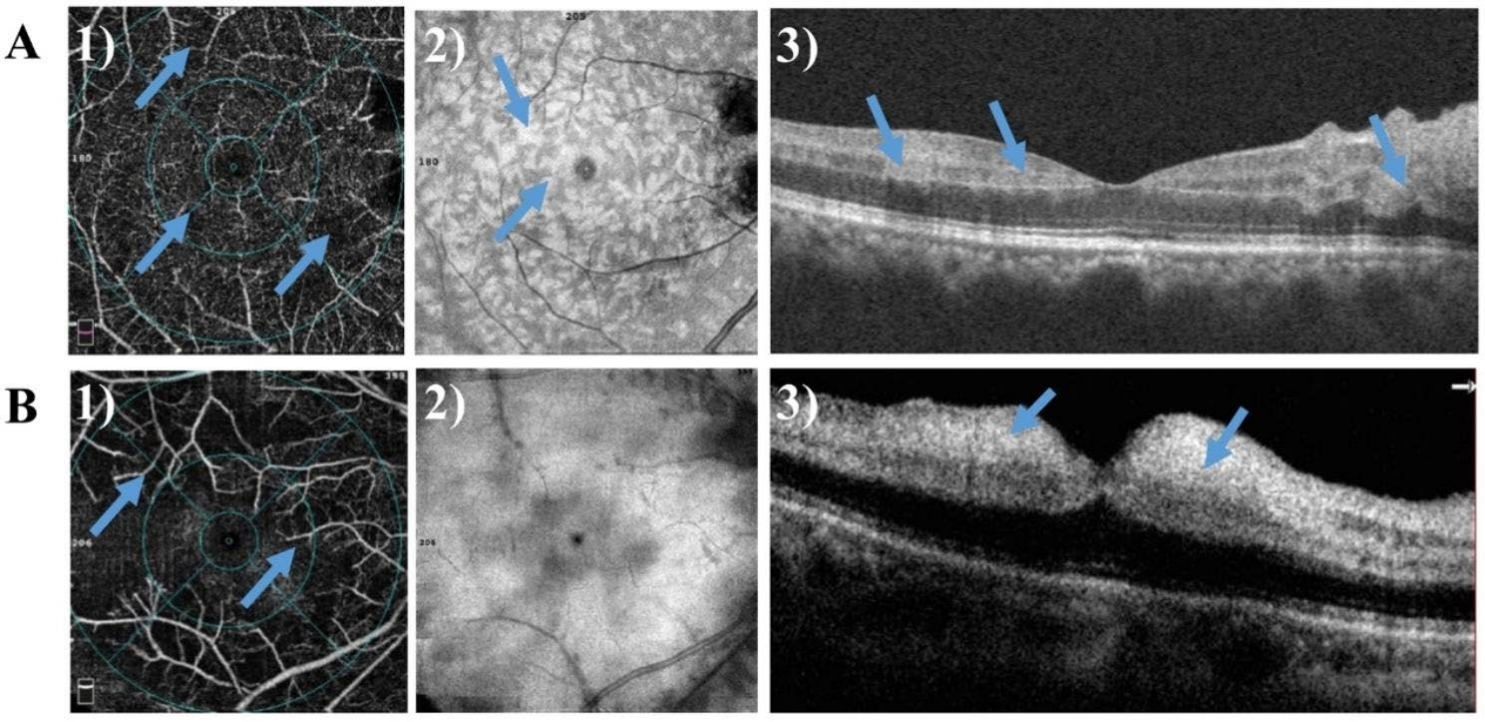
The OCT-A images. **(A)** The OCT-A of PAMM patient. A1). The arrows indicated partial occlusion of terminal arterioles and capillaries; A2). The arrows indicated a fern-like high fluorescence appearance that was more obvious than that of the shallow layer in the deep en face OCT-A image; A3). The arrows indicated hyper-reflectivity and edema in the inner layer in the B-scan OCT-A image. **(B)** The OCT-A of non-PAMM patient. B1). The arrows indicated the retinal branch vessels were broken; B2). The en face OCT-A image demonstrated diffuse hyperreflectivity of the macular retina; B3). The arrows indicated there were macular retinal inner layer edema and ambiguous layers in the B-scan OCT-A image. OCT: optical coherence tomography; OCT-A: optical coherence tomography angiography; PAMM: paracentral acute middle maculopathy

In the PAMM group (Fig. 3A), the density of the superficial and deep macular vessels decreased, and some small terminal arteries and capillaries showed no blood flow signals. The corresponding en face map showed hyperreflective lesions at the occlusion of terminal arterioles. The whole en face image revealed PAMM-like scattered, dotted, lamellar, and fern-like hyperreflective changes, with more significant changes observed for deep vessels than superficial vessels. In the non-PAMM group (Fig. 3B), the density of superficial and deep macular vessels decreased significantly, and most of the branching arteries and veins did not show blood flow signals, with broken branch-like changes. The corresponding en face image demonstrated diffuse hyperreflectivity, and the main vessels of broken branches were submerged in the diffuse high-fluorescence images. Different degrees of hyperreflectivity in the inner retinal layers and retinal thickening were observed on the B-scan images of both the PAMM and non-PAMM groups (Fig. 3).

### Clinical characteristics

**Fig. 4:**
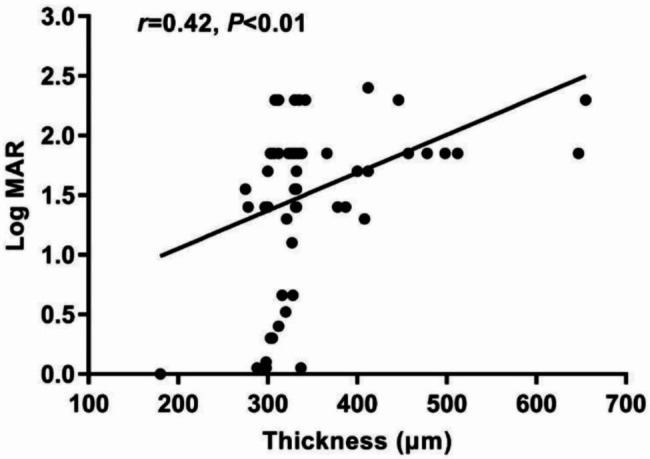
Spearman’s correlation between the LogMAR BCVA and thickness (retinal thickness of the macula). BCVA: best-corrected visual acuity; LogMAR = Log (1/fractional visual acuity)

According to OCT-A images, the CRAO patients were divided into the PAMM group (n = 34) with PAMM-like hyperreflectivity and the non-PAMM group (n = 30) with diffuse hyperreflectivity and extensive retinal edema. There were no significant differences in age, gender, eye type, intraocular pressure, time from onset to treatment, and superficial and deep vascular density between the PAMM and no-PAMM groups (all *P* > 0.05). Compared with the no-PAMM group, the retina of the central macular sulcus in the PAMM group was much thinner (median (IQR): 328.00 (304.50 -332.25) Vs. 352.50 (311.75–420.50), *P* = 0.01). CRAO patients with PAMM had significantly better visual acuity in LogMAR BCVA (median, IQR: 1.48 (0.49–1.85) Vs. 1.85 (1.70–1.96), *P* < 0.01). Detailed data was shown in Table 1. A significant correlation between LogMAR BCVA and macular retinal thickness was found (r = 0.42; *P* < 0.01, Fig. 4).

**Table 1:** Comparison of basic information between the PAMM and no-PAMM groups

Characteristics	PAMM group (n = 34)	No-PAMM group (n = 30)	*P* - value
Demographic characteristics			
Age (years)	57.26 ± 14.38	63.10 ± 9.45	0.06
Male (%)	19 (55.88)	16 (53.33)	0.71
**Clinical characteristics**			
Left eye	22 (64.71)	19 (63.33)	0.63
Intraocular pressure (mmHg)	15.43 ± 3.25	16.72 ± 4.36	0.54
Time from onset to treatment (hours), (median, IQR)	48.00 (20.00–120.00)	53.00 (24.00–76.50)	0.84
LogMAR BCVA, (median, IQR)	1.48 (0.49–1.85)	1.85 (1.70–1.96)	< 0.01
Retinal thickness of the central macular sulcus (µm), (median, IQR)	328.00 (304.50–332.25)	352.50 (311.75–420.50)	0.01
Superficial vascular density (%), (median, IQR)	39.8 (36.3–43.6)	40.5 (38.1–50.6)	0.44
Deep vascular density (%), (median, IQR)	39.9 (36.4–43.3)	42.2 (34.5–50.6)	0.74

BCVA, best-corrected visual acuity; LogMAR: logarithm of the minimum angle of resolution; IQR, interquartile range; PAMM: paracentral acute middle maculopathy

LogMAR = Log (1/fractional visual acuity)

## Discussion

Characterized by partial or complete occlusion of the central retinal artery, CRAO often leads to catastrophic vision loss. It is the ocular equivalent of an ischemic cerebral stroke [14]. PAMM refers to retinal lesions with changes in the inner nuclear layer on SD-OCT [15]. With the advent of OCT-A, PAMM has been considered as vascular pathology resulting from ischemia of deep retinal layers [16]. Various studies have shown that PAMM is related to retinal ischemia [15, 17, 18] and is an accompanying symptom of potential retinal vascular disease, representing activated microglial tissue caused by hypoxia and upregulated inflammatory molecules in an attempt to repair ischemic tissue [19]. B-scan OCT-A has revealed hyperreflective foci dominated by the core layer, with PAMM-like changes disappearing with the thinning of the inner nuclear layer [8, 16, 20]. In this study, patients with CRAO were divided into the PAMM and no-PAMM groups using OCT-A combined with FFA according to the presence or absence of PAMM-like changes. Both groups analyzed visual acuity and related factors in the affected eyes.

Previously study reported PAMM generally had normal fluorescence on FFA [21, 22]. We also found it was difficult to detect PAMM by FFA. In the PAMM group, OCT-A could reveal a lack of blood flow signals in the small terminal retinal arteries in the superficial and deep layers and in successive capillary networks. Browning et al. [23] reported that the appearance of PAMM-like changes might signify mild retinal ischemia. Similarly, we observed many scattered retinal soft exudates in the fundus and PAMM-like hyperreflectivity in the PAMM group. These all indicated retinal ischemia. Therefore, we hypothesized that PAMM-like CRAO might occur due to occlusion a. Unlike the devastating visual impairment of conventional CRAO, it seems to be a self-regulatory mechanism of the body. The small terminal arteries and their connected capillaries regulate vascular resistance by constriction and occlusion, maintaining a constant blood flow in tissues when tissue perfusion pressure changes. OCT-A showed small terminal arteries and capillaries lacking blood flow signal in the PAMM groups, which may be a sign of this mechanism.

In the non-PAMM group, a large area of trunk vessels was observed by FFA and OCT-A, with a broken branch-like absence of blood flow signal and no perfusion area. The increase in macular thickness due to retinal edema was also more significant than that in the other group. Similarly, Abdellah [19] also reported the macular thickness increased in 66.67% of CRAO cases which indicated macular swelling on ischemia. It may be caused by decompensation loss due to poor perfusion. As observed in the non-PAMM group, all cells in the inner layer of the retina underwent acute swelling in severe retinal ischemia, resulting in hyperreflectivity of the entire inner layer of the retina by B-scan OCT-A, which was a relatively severe manifestation of ischemic damage. A recent study from Feucht reported the inner retinal layers hyperreflectivity in all CRAO cases [24]. But it did not indicate whether these CRAO case had the accompanying symptom of PAMM.

Similar to the previous report, we found the retinal thickness of the central macular sulcus positively correlated with the LogMAR BCVA in the current study [25]. We also found the retinal of the central macular sulcus in the no-PAMM group was much thicker. More severe macular edema indicated a higher degree of retinal ischemia, thus leading to worse visual acuity. Ahn S et al. also reported that the initial macular edema in patients with CRAO was significantly related to final BCVA, though the initial BCVA was not record in their study [26]. These results suggested that the degree of visual function impairment mainly depended on the severity of CRAO. Macular retinal thickness may be a manifestation of the degree of ischemia. Furthermore, the final central macular thickness was related to a poor visual prognosis in retinal artery occlusion [26, 27]. However, no significant differences in the superficial and deep vascular density were observed between the two groups, which may be caused by the machine’s inability to recognize slow flow. PAMM-like changes might be an essential indicator for distinguishing the degree of impairment. PAMM might be due to hypoxia, and the blood vessels were not completely blocked, leading to hypoxia. However, in CRAO patients without PAMM, full-thickness retinal edema and complete lack of oxygen (anoxia) to the cells might result in poorer visual acuity [28].

The present study has several limitations. Firstly, it is a single-center and retrospective study. Although consecutive patients were screened for eligibility, selection bias can’t be excluded. Secondly, the sample size was small, and the results need to be confirmed furtherly by a more extensive and prospective study. Lastly, due to the small sample size, CRAO was not further typed based on OCT-A.

## Conclusion

The present study shows that CRAO patients with PAMM experience less severe impairment of visual function than CRAO patients without PAMM and that there are unique manifestations of OCT-A and FFA in the PAMM group. There was a significant correlation between LogMAR BCVA and macular retinal thickness. The importance of this study was evaluating the degree of visual function damage due to CRAO based on the presence of PAMM, with apparent differences in performance by OCT-A and FFA and possible implications for future treatments.

## Data Availability

The data materials were obtained from reasonable request to the corresponding author. All data and materials were unpublished.

## Abbreviations

BCVA: Best-corrected visual acuity
CRAO: Central retinal artery occlusion
FFA: Fundus fluorescein angiography
IQR: Interquartile range
LogMAR: logarithm of the minimum angle of resolution
MAR: Logarithmic angle of resolution
OCT-A: Optical coherence tomography angiography
PAMM: Paracentral acute middle maculopathy
SD-OCT: Spectral domain optical coherence tomography
